# Effect of pasireotide on glucose- and growth hormone-related biomarkers in patients with inadequately controlled acromegaly

**DOI:** 10.1007/s12020-016-0895-8

**Published:** 2016-02-23

**Authors:** Herbert A. Schmid, Thierry Brue, Annamaria Colao, Mônica R. Gadelha, Ilan Shimon, Karen Kapur, Alberto M. Pedroncelli, Maria Fleseriu

**Affiliations:** Novartis Pharma AG, Postfach, Basel, Switzerland; Centre National de la Recherche Scientifique, and Assistance Publique-Hôpitaux de Marseille, Hôpital de la Conception, Aix-Marseille University, Marseille, France; Università Federico II di Napoli, Naples, Italy; Hospital Universitário Clementino Fraga Filho, Universidade Federal do Rio de Janeiro, Rio de Janeiro, Brazil; Institute of Endocrinology and Metabolism, Rabin Medical Center, and Sackler School of Medicine, Tel-Aviv University, Petah Tikva, Israel; Northwest Pituitary Center, Oregon Health & Science University, Portland, OR USA

**Keywords:** PAOLA, Pasireotide, HbA_1c_, Glucose, Hyperglycaemia, Acromegaly

## Abstract

The purpose of this study was to gain more insight into the mechanism of action of pasireotide in patients who completed the PAOLA study. PAOLA was a 24-week, Phase III, randomized, three-arm study of pasireotide LAR 40 and 60 mg versus octreotide LAR 30 mg or lanreotide Autogel 120 mg in patients with inadequately controlled acromegaly. The current work was a planned exploratory objective of the PAOLA study that evaluated changes in levels of growth hormone (GH), insulin-like growth factor 1 (IGF-1), IGF-binding proteins (IGFBP-2, IGFBP-3), glycated haemoglobin (HbA_1c_) and fasting plasma glucose (FPG) in each treatment arm. Responders to pasireotide LAR (mean GH levels <2.5 μg/L and normal IGF-1 levels at 24 weeks) had lower GH and IGF-1 levels at baseline (GH 5.1 ng/mL, IGF-1 519 ng/mL) than non-responders (GH 7.9 ng/mL, IGF-1 672 ng/mL). Frequency of hyperglycaemia after pasireotide treatment was similar in responders and non-responders and depended more on the baseline FPG level. 47 % of all patients treated with pasireotide LAR (40 or 60 mg) did not receive antidiabetic medication at any time during this study. This is the first study to evaluate the treatment effect of pasireotide on key hormonal and glycaemic biomarkers and to identify potential predictors of pasireotide-associated hyperglycaemia. Pre-treatment glucose status may be predictive of the development of pasireotide-associated hyperglycaemia. A large subset of patients with acromegaly does not experience major disturbances in glucose homeostasis while receiving pasireotide LAR.

**Electronic supplementary material:**

The online version of this article (doi:10.1007/s12020-016-0895-8) contains supplementary material, which is available to authorized users.

## Introduction

Medical therapy targeting somatostatin receptors is a mainstay treatment of disorders associated with hormone hypersecretion, such as acromegaly [1].

Five somatostatin receptor subtypes (SSTR1–5) have been identified [2]. In acromegaly, both first-generation somatostatin analogues, octreotide and lanreotide, exert their pharmacological effects mainly by binding to SSTR2 and to a lesser extent to SSTR5 [3], which are both expressed by most growth hormone (GH)-secreting pituitary adenomas [4]. The excess GH and insulin-like growth factor 1 (IGF-1) levels associated with acromegaly are suppressed in most patients following treatment with somatostatin analogues [5]. However, despite the clinical success of octreotide and lanreotide therapy in the treatment of acromegaly, approximately half of patients remain inadequately controlled [5] and are exposed to the deleterious effects of hormone hypersecretion, including significant co-morbidities, notably diabetes mellitus, increased mortality risk, and diminished quality of life [1]. Thus, alternative therapeutic options are needed.

Pasireotide, a multireceptor-targeted somatostatin analogue, has a high binding affinity for SSTR1, 2, 3, and 5 (exhibiting a 39-fold higher binding affinity for SSTR5 compared with octreotide [6]), with more profound suppression of GH and IGF-1 than octreotide [7, 8]. In a randomized, Phase III study in medically naïve patients with acromegaly, pasireotide LAR demonstrated superior efficacy over octreotide LAR [9]. More recently, in the PAOLA study, pasireotide LAR provided superior efficacy versus continued treatment with octreotide LAR or lanreotide Autogel (control group) in patients with inadequately controlled acromegaly [10]. In both of these studies, the safety profile of pasireotide LAR was generally similar to that of first-generation somatostatin analogues, except for the frequency and degree of hyperglycaemia.

Here, we report the results of a planned exploratory objective of the PAOLA study. To gain more insight into the mechanism of action of pasireotide in patients with acromegaly inadequately controlled with first-generation somatostatin analogues, changes in the clinically established efficacy biomarkers GH and IGF-1, as well as other biomarkers (IGF-binding protein [IGFBP]-2/3, glucose and glycated haemoglobin [HbA_1c_]), were assessed after pasireotide LAR treatment. Additionally, baseline levels of these markers were evaluated as potential predictors of clinical outcome.

## Subjects and methods

### Patient population

Male and female patients aged ≥18 years with inadequately controlled acromegaly, defined as mean GH > 2.5 μg/L *and* IGF-1 > 1.3 times the sex- and age-adjusted upper limit of normal (ULN), were enrolled in this study. All patients had received treatment with octreotide LAR 30 mg or lanreotide Autogel 120 mg for at least 6 months prior to screening. Patients could have received prior surgery [10].

### Study design

PAOLA was a prospective, multicentre, randomized, parallel-group study. The study design has been described more fully elsewhere [10]. Briefly, after a 4-week screening period, patients were randomized to receive double-blind pasireotide LAR 40 mg every 28 days for 24 weeks, or double-blind pasireotide LAR 60 mg every 28 days for 24 weeks, or to continue on the same treatment with open-label octreotide LAR 30 mg or lanreotide Autogel 120 mg every 28 days for 24 weeks (active control group). Transient dose decreases were permitted for tolerability issues in all treatment arms.

### Study objectives

In the PAOLA study, the primary objective was to compare the proportion of patients achieving biochemical control, defined as mean GH levels <2.5 µg/L and normalization of sex- and age-adjusted IGF-1 at 24 weeks, with pasireotide LAR 40 mg and pasireotide LAR 60 mg separately versus continuing the same treatment with octreotide LAR 30 mg or lanreotide Autogel 120 mg. The main secondary endpoint was the proportion of patients achieving normalization of sex- and age-adjusted IGF-1 at 24 weeks. The outcomes are fully described elsewhere [10].

The objectives of the current work were to assess changes in hormonal biomarkers (GH, IGF-1, IGFBP-2, and IGFBP-3) and glucose homeostasis biomarkers (fasting plasma glucose [FPG] and HbA_1c_) over time by treatment, GH/IGF-1 response status, and the use of antidiabetic medication.

### Biochemical assessments

Blood samples for assessment of total IGF-1 were taken at the same visits as for the assessment of mean GH level, which was at screening, baseline, week 12, and week 24 (study completion). In addition, the mean IGF-1 level was also assessed at week 4. All blood samples were taken before the administration of study drug. Samples were analysed as follows: GH was assessed as the mean of an average of five individual measurements taken pre-dose at 0, 30, 60, 90, and 120 min within a 2-h time period after 1 h at rest at the hospital and was measured using the Siemens Immulite 2000 S/N 1832 assay by a central contract research organization (Quest Diagnostics Clinical Trials, Valencia, CA, USA). IGF-1 and IGFBP-3 were analysed from individual serum samples using the Siemens Immulite 2000 S/N 1832 IGF-1 assay and the DPC Immulite 2000 IGFBP-3 assay, respectively (Quest Diagnostics Clinical Trials, Valencia, CA, USA); IGFBP-2 was analysed in serum using the R&D Systems IGFBP-2 enzyme-linked immunosorbent assay (ELISA) kit (Cat#DY674; BioAgilytix Labs, Durham, NC, USA); and FPG and HbA_1c_ were centrally analysed (Quest Diagnostics Clinical Trials, Valencia, CA, USA) by spectrophotometry using an Olympus AU 640/2700/5400 analyser and by high-performance liquid chromatography (HPLC) using a TOSOH G7/G8 automated HPLC analyser, respectively.

### Statistical analyses

The PAOLA study was not powered to assess specific biomarker-related hypotheses, so the statistical analyses of biomarker data should be considered as hypothesis driven and exploratory in nature. Analyses were conducted on patients who had available samples and who completed this study. Time profiles of GH, IGF-1, IGFBP-2, IGFBP-3, FPG, and HbA_1c_ levels were analysed together and separately in relevant subgroups of patients as follows: patients who were responders (GH < 2.5 μg/L and normal IGF-1) or non-responders (GH ≥ 2.5 μg/L and/or IGF-1 > ULN) at week 24; patients who did not receive antidiabetic medication at any time during the study; patients who were receiving antidiabetic medication at baseline; patients who initiated antidiabetic medication during the study (i.e. post-baseline); and patients with baseline FPG > 100 and ≤100 mg/dL. Hyperglycaemia was defined as one post-baseline FPG measurement of ≥126 mg/dL or receiving antidiabetic medication at any time during this study.

For biomarkers showing evidence of a skewed distribution, summaries of natural-log-transformed values, geometric means or medians were reported instead of arithmetic means. GH, IGF-1, IGFBP-2 and IGFBP-3 values reported below the lower limit of quantitation (LLOQ) or above the upper limit of quantitation (ULOQ) were imputed by LLOQ/2 or 0 and by ULOQ or the maximal value below the ULOQ, respectively. Statistical analyses were performed by Novartis using R version 3.0.1 or later and SAS version 9.2 or later.

## Results

### Patient population

The patient population has been described in more detail previously [10]. Briefly, a total of 198 patients were randomized: 65 each to pasireotide LAR 40 and 60 mg, and 68 to continued treatment with octreotide LAR 30 mg or lanreotide Autogel 120 mg (active control). Patient demographics, characteristics and disease history were generally similar across the three treatment groups at baseline. In total, 59 (90.8 %), 57 (87.7 %), and 65 (95.6 %) patients in the pasireotide LAR 40 mg, pasireotide LAR 60 mg, and active control groups completed the 24-week study, respectively.

### Changes in GH, IGF-1, IGFBP-2, and IGFBP-3 levels

**Fig. 1:**
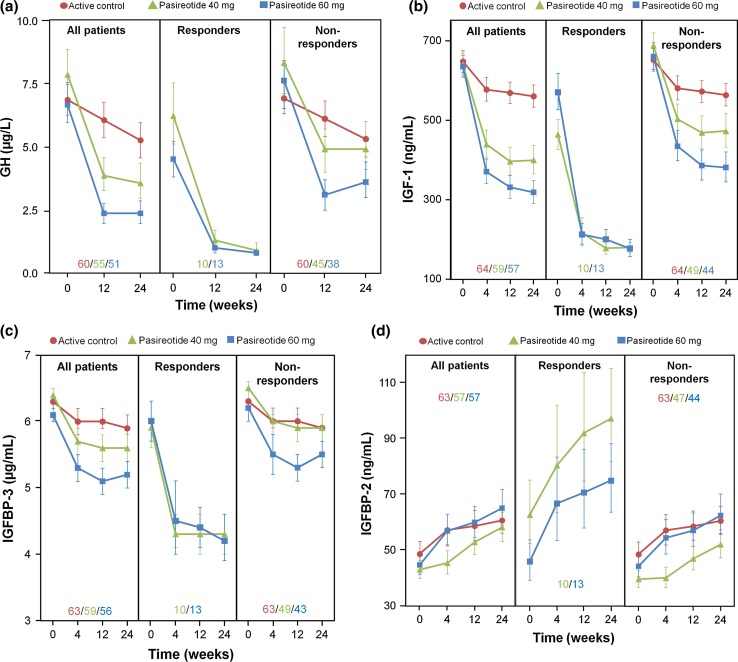
Effect of pasireotide LAR and active control on **a** GH, **b** IGF-1, **c** IGFBP-3, and **d** IGFBP-2 levels during 24 weeks of treatment in all patients, responders, and non-responders. Data presented as geometric mean and 68 % CI. *Inset values* represent patient numbers at 24 weeks

A decrease from baseline in GH, IGF-1, and IGFBP-3 levels was seen with both pasireotide and active control, with the magnitude of decrease being higher in the pasireotide LAR 40 and 60 mg groups. Mean baseline values for GH, IGF-1, and IGFBP-3 were similar between pasireotide LAR and active control; however, baseline values for these parameters were lower among responders compared with non-responders to pasireotide (Table 1; Fig. 1). The overall median decrease from baseline to week 24 in GH and IGF-1 levels was numerically greater in the pasireotide LAR 60 mg group compared with the 40 mg group, with this dose dependency being most apparent among non-responders. Notably, decreases in both GH and IGF-1 among non-responders were more marked in both pasireotide arms compared with active control. For IGFBP-2, the median (interquartile range [IQR]) percentage changes from baseline in IGFBP-2 levels for pasireotide LAR 40 mg (27 % [73 %]), 60 mg (36 % [86 %]) and active control (25 % [85 %]) were similar at week 24 (Fig. 1). However, for pasireotide LAR 40 and 60 mg, respectively, the median (IQR) percentage changes in IGFBP-2 levels from baseline to week 24 were 66 % (41 %) and 37 % (72 %) for treatment responders versus 26 % (80 %) and 33 % (91 %) for non-responders.

**Table 1:** Effects of pasireotide and active control on GH, IGF-1, and IGFBP-3 after 24 weeks of treatment in all patients

	*N* ^a^	Baseline geometric mean for all patients (95 % CI)	Baseline geometric mean for responders (95 % CI)	Baseline geometric mean for non-responders (95 % CI)	Week 24 geometric mean for all patients (95 % CI)	Median (IQR) change from baseline to week 24 for all patients (%)
Pasireotide 40 mg						
GH (ng/mL)	59	7.9 (6.0–10.3)	6.2 (4.2–9.0)	8.3 (6.1–11.4)	3.6 (2.5–5.3)	−51.4 (39.8)
IGF-1 (ng/mL)	59	640.7 (586.1–700.3)	462.1 (391.6–545.3)	684.8 (625.2–750.1)	400.1 (335.3–477.3)	−38.6 (54.9)
IGFBP-3 (µg/mL)	59	6.4 (6.1–6.6)	5.9 (5.4–6.6)	6.5 (6.2–6.7)	5.6 (5.2–5.9)	−13.5 (22.1)
Pasireotide 60 mg						
GH (ng/mL)	57	6.7 (5.2–8.7)	4.5 (3.3–6.1)	7.6 (5.5–10.4)	2.4 (1.7–3.5)	−61.3 (43.0)
IGF-1 (ng/mL)	57	635.7 (582.5–693.8)	567.9 (484.1–666.3)	657.3 (593.7–727.6)	319.1 (266.1–382.6)	−48.9 (42.2)
IGFBP-3 (µg/mL)	57	6.1 (5.9–6.4)	6.0 (5.5–6.6)	6.2 (5.9–6.5)	5.2 (4.8–5.6)	−13.2 (21.1)
Pasireotide 40/60 mg						
GH (ng/mL)	116	7.3 (6.0–8.8)	5.1 (4.0–6.6)	7.9 (6.4–9.9)	3.0 (2.3–3.9)	−55.8 (45.6)
IGF-1 (ng/mL)	116	638.2 (599.8–679.2)	519.2 (460.1–586.0)	671.6 (627.7–718.7)	358.0 (315.1–406.8)	−44.3 (48.7)
Active control						
GH (ng/mL)	65	6.9 (5.7–8.2)	–	–	5.3 (4.1–6.8)	−14.7 (52.7)
IGF-1 (ng/mL)	65	648.5 (594.3–707.7)	–	–	561.1 (508.2–619.5)	−7.4 (32.3)
IGFBP-3 (µg/mL)	65	6.3 (6.1–6.6)	–	–	5.9 (5.6–6.2)	−5.2 (18.1)

*CI* confidence interval, *IQR* interquartile range

^a^Number of patients with baseline measurements

### Changes in glycaemic biomarkers

**Fig. 2:**
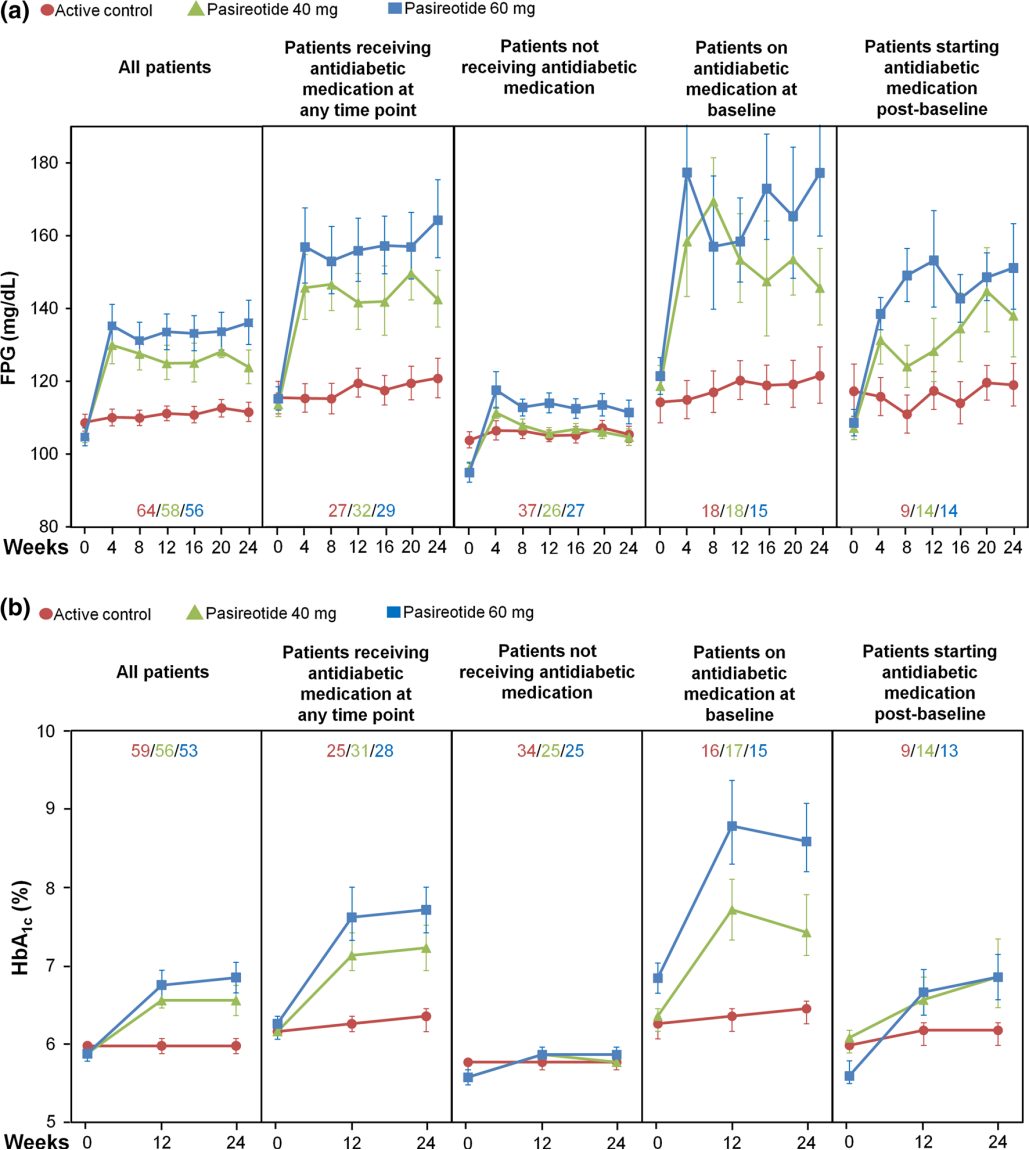
Effect of pasireotide LAR and active control on **a** FPG and **b** HbA_1c_ levels in all patients, patients receiving antidiabetic medication at any time point, patients not receiving antidiabetic medication, patients receiving antidiabetic medication at baseline, and patients starting antidiabetic medication post-baseline. Data presented as geometric mean and 68 % CI. *Inset values* represent patient numbers at 24 weeks

In patients receiving pasireotide LAR 40 and 60 mg, respectively, 44 and 49 % did not receive antidiabetic medication during this study, 31 and 26 % were receiving antidiabetic medication at study entry, and 25 and 25 % of patients initiated antidiabetic medication during this study. Patients who received antidiabetic medication during pasireotide LAR treatment had higher baseline mean FPG and HbA_1c_ levels (Fig. 2) and more variable FPG levels (Supplementary Fig. 1) than patients who received no antidiabetic medication.

### Effect of baseline glucose status on glucose and HbA_1c_ levels during treatment

**Fig. 3:**
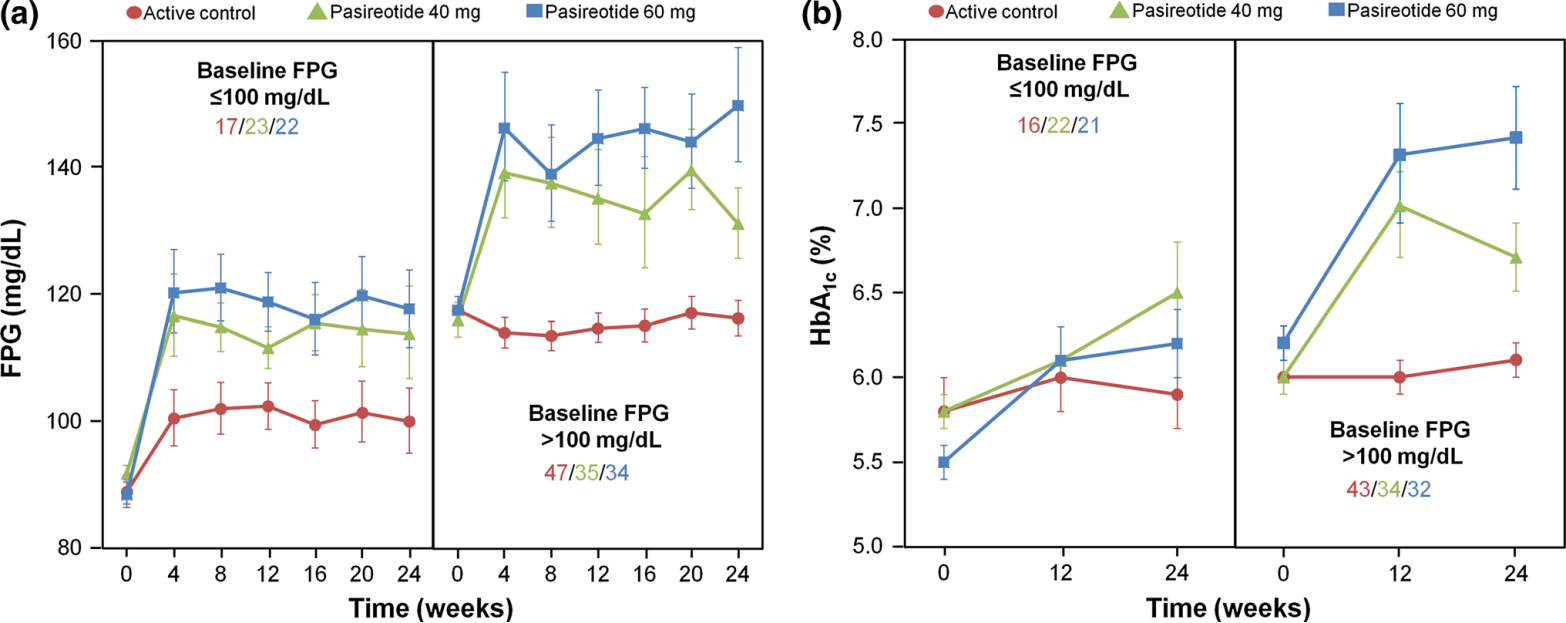
Effect of pasireotide LAR and active control on **a** FPG and **b** HbA_1c_ in patients with baseline FPG ≤ 100 or >100 mg/dL. Data presented as geometric mean and 68 % CI. *Inset values* represent patient numbers at 24 weeks

Analysis of all patients revealed that those with baseline FPG > 100 mg/dL experienced higher values of FPG and HbA_1c_ after treatment with pasireotide LAR than patients with baseline FPG levels ≤100 mg/dL (Fig. 3; Table 2).

**Table 2:** Effect of pasireotide and active control on glucose and HbA_1c_ levels after 24 weeks of treatment, stratified according to baseline glucose status

	*N* ^a^	Mean baseline FPG (mg/dL) (95 % CI)	Week 24 mean FPG (mg/dL) (95 % CI)	Median (IQR) change from baseline to week 24 (%)	*n* ^b^	Mean baseline HbA_1c_ (%)	Week 24 mean HbA_1c_ (%) (95 % CI)	Median (IQR) change from baseline to week 24 (%)
Pasireotide 40 mg								
FPG ≤ 100 mg/dL	24	91.8 (89.2–94.5)	113.8 (100.1–129.4)	14.9 (14.1)	24	5.8 (5.6–6.0)	6.5 (5.9–6.4)	5.6 (11.1)
FPG > 100 mg/dL	35	115.9 (110.5–121.4)	131.0 (120.3–142.7)	8.1 (24.1)	34	6.0 (5.8–6.2)	6.7 (6.3–7.1)	9.4 (11.1)
Pasireotide 60 mg								
FPG ≤ 100 mg/dL	23	88.4 (84.4–92.6)	117.7 (106.1–130.5)	30.7 (33.9)	23	5.5 (5.3–5.6)	6.2 (5.8–6.6)	10.5 (13.7)
FPG > 100 mg/dL	34	117.4 (113.3–121.8)	149.6 (132.6–168.7)	22.4 (26.7)	33	6.2 (5.9–6.5)	7.4 (6.8–8.0)	12.1 (21.0)
Active control								
FPG ≤ 100 mg/dL	18	88.9 (85.2–92.8)	100.0 (90.3–110.8)	7.0 (9.6)	17	5.8 (5.6–6.1)	5.9 (5.6–6.2)	−1.7 (8.8)
FPG > 100 mg/dL	47	117.4 (113.1–121.8)	116.2 (110.7–121.9)	−2.26 (12.3)	44	6.0 (5.9–6.2)	6.1 (5.9–6.2)	0 (5.2)

^a^Number of patients with baseline measurements for FPG

^b^Number of patients with baseline measurements for HbA_1c_

**Fig. 4:**
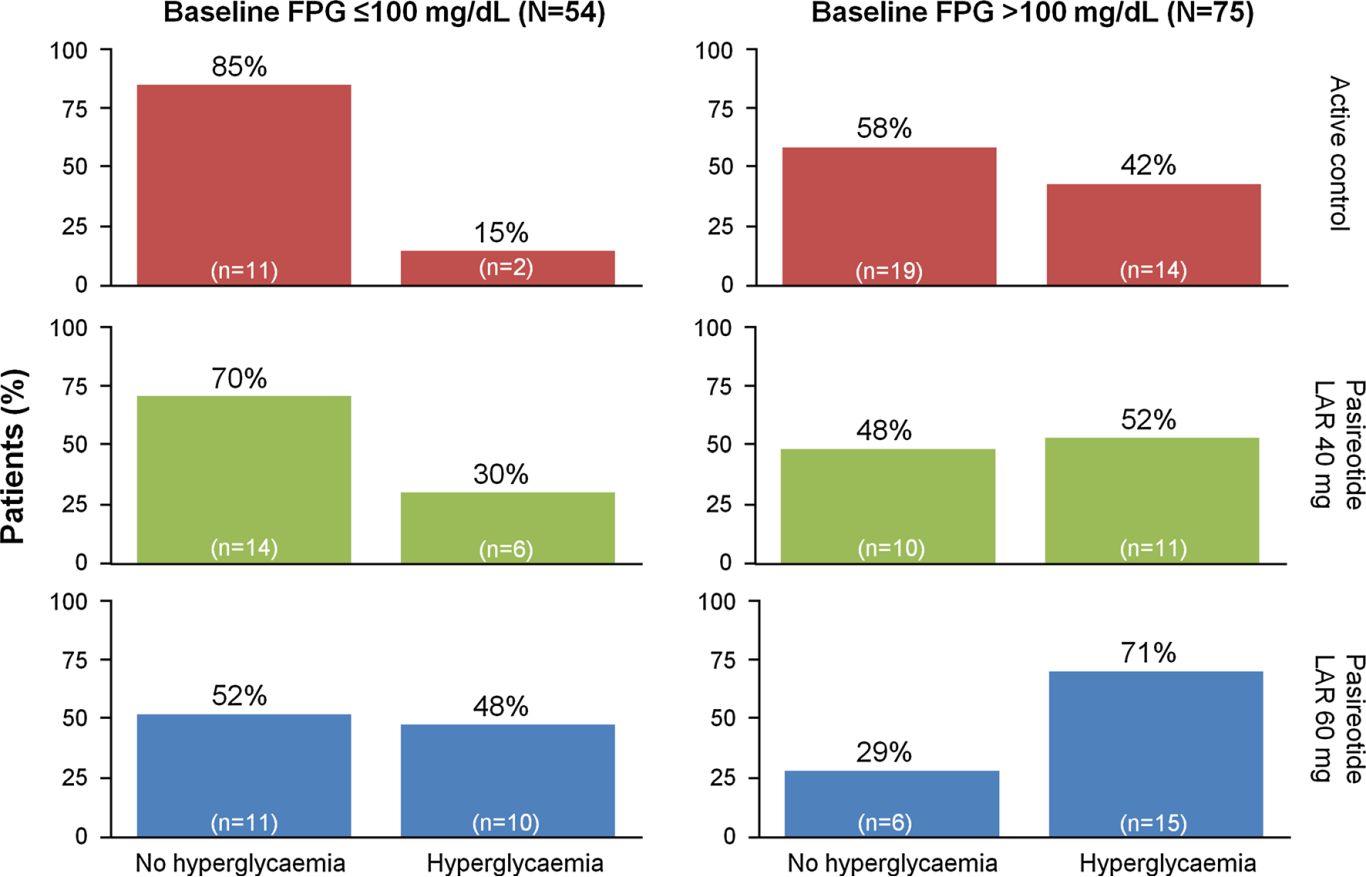
Percentage of patients developing hyperglycaemia (post-baseline FPG ≥ 126 mg/dL or receiving antidiabetic medication post-baseline) during treatment with pasireotide LAR or active control, stratified by baseline FPG levels ≤ 100 or >100 mg/dL, in patients without baseline antidiabetic medication

Among patients not receiving antidiabetic medication at baseline and with baseline FPG ≤ 100 mg/dL, 30 % (6/20) and 48 % (10/21) developed hyperglycaemia (FPG > 126 mg/dL) during treatment with pasireotide LAR 40 mg and 60 mg, respectively. In contrast, 52 % (11/21) and 71 % (15/21) of patients with baseline FPG > 100 mg/dL developed hyperglycaemia during treatment (Fig. 4).

In the pasireotide LAR 40 mg treatment group, geometric mean baseline FPG was 104.1 mg/dL (95 % CI 91.9–117.8) and 105.6 mg/dL (95 % CI 101.0–110.5) for responders and non-responders, respectively, which increased to 124.4 mg/dL (95 % CI 115.0–134.6) and 123.8 mg/dL (95 % CI 113.4–135.2) at week 24 (median [IQR] percentage change from baseline, 19.4 % [23.3 %] and 10.7 % [16.5 %]). For the 60 mg dose of pasireotide, responders and non-responders, respectively, had mean baseline FPG levels of 100.7 mg/dL (95 % CI 88.3–114.7) and 106.0 mg/dL (95 % CI 101.1–111.1), which increased to 121.2 mg/dL (95 % CI 100.9–145.6) and 141.0 mg/dL (95 % CI 127.5–155.8) at week 24 (median [IQR] percentage change from baseline, 19.4 % [36.5 %] and 24.5 % [27.0 %]).

The effect of pasireotide LAR treatment on HbA_1c_ was similar for responders and non-responders: the median (IQR) percentage changes from baseline to week 24 for responders were 9.2 % (19.1 %) and 10.6 % (7.7 %) for pasireotide LAR 40 mg and 60 mg, respectively, and 6.7 % (12.1 %) and 12.1 % (20.5 %) for non-responders. In addition to these findings, responder rates were found to be very similar in patients who did (21 % [*n* = 13]) and did not (19 % [*n* = 10]) receive antidiabetic medication.

## Discussion

GH and IGF-1 are both closely linked to glycaemic status. GH suppresses insulin-mediated glucose uptake and enhances hepatic gluconeogenesis, while IGF-1, in addition to enhancing insulin action, also suppresses GH secretion [11]. These antagonistic effects act to modulate responsiveness to insulin. Sustained or excess GH secretion is associated with insulin resistance in both physiological (in healthy individuals during exercise and sleep) and pathological (acromegaly) states [12, 13], while the association between IGF-1 and insulin resistance is complex and remains less well understood.

IGF-1 (and, indirectly, GH) is regulated by a family of six homologous IGFBPs. Levels of IGFBP-3, the major binding protein for IGF-1, increase in response to increased levels of GH and IGF-1 [14, 15]. IGFBP-3 was once considered to be a potential biochemical marker of excess GH in patients with suspected acromegaly [16]. A relationship between increased levels of IGFBP-3 and hyperglycaemia has been suggested. In murine models, IGFBP-3 inhibits insulin-stimulated glucose uptake [17], while over-expression of IGFBP-3 in male transgenic mice results in fasting hyperglycaemia, impaired glucose tolerance, and insulin resistance [18]. In the current analysis, dose-dependent reductions in GH, IGF-1, and IGFBP-3 levels were observed with pasireotide LAR, with levels of all three markers remaining consistently suppressed during the treatment period. When these findings were stratified according to whether patients were responders or non-responders to pasireotide LAR treatment, the responder group had lower GH levels and lower IGF-1 levels at baseline than the non-responder group. This finding is consistent with a previous population pharmacodynamic analysis of octreotide in patients with acromegaly, in which high baseline GH levels were associated with poorer responses to octreotide treatment [19]. For IGFBP-3, baseline levels were similar in both responders and non-responders.

Glucose homeostasis was relatively undisturbed throughout the treatment period in those patients receiving continued therapy with either octreotide or lanreotide. By contrast, there was a rapid initial increase in mean glucose and HbA_1c_ levels following treatment with pasireotide LAR, which subsequently plateaued, remaining stable to 24 weeks. This finding is consistent with previous Phase III study observations in which pasireotide-associated elevations in glucose levels typically plateaued after approximately 2–3 months of pasireotide treatment in patients with acromegaly [20] and Cushing’s disease [21–23]. Patients with baseline glucose levels >100 mg/dL experienced higher levels of glucose and HbA_1c_ after treatment with pasireotide LAR compared with patients with normoglycaemia at baseline. Although results vary, the data suggest that baseline glucose status is a potential predictive factor for the development of hyperglycaemia during pasireotide LAR treatment.

Patients who received antidiabetic medication during pasireotide LAR treatment had higher baseline mean glucose and HbA_1c_ levels than patients who received no antidiabetic medication. Notably, approximately half of patients treated with pasireotide LAR did not receive antidiabetic medication at any time during this study. This finding suggests that a substantial proportion of patients with acromegaly do not experience disturbances in glucose homeostasis during pasireotide LAR treatment that warrants the initiation of antidiabetic medication.

Given the physiological role of natural somatostatin, as well as the SSTR binding profile of pasireotide [24], disturbances in glucose metabolism are not unexpected during treatment with pasireotide. Endocrine cells of the pancreas consist of α-, β-, and δ-cells, which secrete glucagon, insulin, and somatostatin, respectively, in response to changes in blood glucose. Insulin and glucagon are antagonistic hormones that regulate glucose uptake and metabolism, while localized release of somatostatin suppresses secretion of insulin and glucagon. In humans, glucagon-producing α-cells predominantly express SSTR2 [25], whereas SSTR5 and SSTR2 are found mainly on insulin-producing β-cells [26]. As pasireotide binds with higher affinity to SSTR5 than to SSTR2 [24], insulin secretion is substantially reduced while glucagon secretion is less markedly suppressed, resulting in an overall increase in glucose levels. Preclinical observations showed that pasireotide and octreotide suppressed insulin secretion to a similar degree, whereas pasireotide was a weaker inhibitor of glucagon secretion than octreotide [27]. Indeed, the SSTR5:SSTR2 activation ratio has been hypothesized to be the main driver of pasireotide-induced hyperglycaemia [27]. Schmid and Brueggen showed that co-administration of octreotide with pasireotide in rats negated the hyperglycaemia seen with pasireotide alone, implying that strong activation of SSTR2 by octreotide was sufficient to restore normoglycaemia [27].

The mechanism of pasireotide-induced hyperglycaemia has been explored in two studies conducted in healthy human volunteers. Henry et al. reported that twice-daily subcutaneous administration of 600 or 900 µg pasireotide significantly decreased plasma levels of insulin, glucagon-like peptide 1 (GLP-1), and glucose-dependent insulinotropic polypeptide [28]. Glucagon secretion was only minimally affected, and insulin sensitivity was unaffected. The effects of pasireotide on insulin (via SSTR5) and glucagon secretion (via SSTR2) in this study were consistent with the SSTR binding profile of pasireotide. In the second study, the incretin-based antihyperglycaemic agents liraglutide (a GLP-1 agonist) and vildagliptin (a dipeptidyl peptidase 4 [DPP-4] inhibitor) were shown to effectively ameliorate hyperglycaemia when co-administered with pasireotide [29].

A similar finding has recently been reported for metformin-based antidiabetic therapy. In a sub-analysis of a 12-month, Phase III, randomized study in medically naïve patients with acromegaly [30], only those pasireotide patients who initiated antidiabetic medication at some point during the study, and not at baseline, were analysed (*n* = 57). In line with its role as first-line medical therapy for glycaemic management, metformin was the most commonly initiated antidiabetic medication. Metformin monotherapy (*n* = 24) or in combination with another oral antidiabetic medication (*n* = 19) was found to be effective in controlling (HbA_1c_ < 7 %) pasireotide-associated hyperglycaemia. Although metformin exerts its therapeutic effect mainly by reducing hepatic glucose production, it also reduces DPP-4 activity and increases GLP-1 secretion, which may be significant in the current context [31]. Taken together with the aforementioned studies, the role of the incretin system affecting insulin secretion is strongly implicated in the mechanism of action of pasireotide.

Endogenous processes may also potentially reduce the impact of pasireotide-induced hyperglycaemia. IGFBP-2 is a regulator of IGF-1, blocking the ability of the latter to bind to its receptor [32, 33], and it is also capable of modulating cellular processes independently of IGF-1 binding [34]. Although the physiological role of IGFBP-2 is not well defined, over-expression of IGFBP-2 in a transgenic mouse model was associated with improved insulin sensitivity compared with wild type [35]. Although data from the current analysis showed that responders to pasireotide LAR had increased levels of IGFBP-2 during treatment compared with non-responders, the effect of pasireotide LAR on levels of glycaemic biomarkers was found to be similar in both responders and non-responders. Whether or not elevated levels of IGFBP-2 confer increased insulin sensitivity, which may offset the effects of pasireotide LAR on glucose homeostasis in this setting, remains unknown. It is possible that the relatively short-term duration of the primary study (6 months) was an insufficient timeframe to observe such a phenomenon.

## Conclusion

Pasireotide LAR has demonstrated superior efficacy over octreotide LAR in medically naïve patients with acromegaly and, in the PAOLA study, in patients with uncontrolled acromegaly versus continued treatment with octreotide LAR or lanreotide Autogel. In both studies, a majority of patients experienced elevations in blood glucose levels with pasireotide treatment. Owing to the mechanism of action of pasireotide, elevated blood glucose despite improved efficacy is not unexpected.

Nevertheless, approximately half of patients with inadequately controlled acromegaly who completed the 24-week PAOLA study did not experience disturbances in glucose homeostasis during pasireotide LAR treatment that warranted the initiation of antidiabetic medication.

Responders to pasireotide LAR (mean GH levels <2.5 μg/L and normalized IGF-1 levels) had lower GH and IGF-1 levels at baseline than non-responders. The effect of pasireotide LAR on blood glucose levels was similar in both responders and non-responders at week 24 and depended on the baseline glucose level.

Hyperglycaemia after pasireotide LAR treatment was observed in some patients who were normoglycaemic at baseline, but the effect was more pronounced in patients with pre-existing hyperglycaemia. Pre-treatment glucose status may thus be predictive of the development of pasireotide-associated hyperglycaemia and could play a future role in selecting appropriate or individualized therapy in patients with acromegaly.

## Electronic supplementary material

Below is the link to the electronic supplementary material.

Supplementary material 1 (DOCX 143 kb)
