# Supplementary material for: Effect of pasireotide on glucose- and growth hormone-related biomarkers in patients with inadequately controlled acromegaly
Source: Endocrine. 2016 Feb 23;53:210–9. doi: 10.1007/s12020-016-0895-8 (PMC4901125; doi:10.1007/s12020-016-0895-8)
Supplement: Supplementary file 1 — Supplementary material 1 (DOCX 143 kb) [file 12020_2016_895_MOESM1_ESM.docx]

**Effect of pasireotide on glucose- and growth-hormone-related biomarkers in patients with inadequately controlled acromegaly**

Herbert A Schmid, Thierry Brue, Annamaria Colao, Mônica R Gadelha, Ilan Shimon, Karen Kapur, Alberto M Pedroncelli, and Maria Fleseriu

**Supplementary material**

**Supplementary Figure 1. Longitudinal change in glucose from baseline of individual patients following treatment with pasireotide LAR or active control stratified by antidiabetic medication status**
